# Supplementary material for: Association between oral microbiome and seven types of cancers in East Asian population: a two-sample Mendelian randomization analysis
Source: Front Mol Biosci. 2023 Nov 21;10:1327893. doi: 10.3389/fmolb.2023.1327893 (PMC10702768; doi:10.3389/fmolb.2023.1327893)
Supplement: Supplementary file 1 [file DataSheet1.ZIP › Supplementary Materials/MR plots of saliva/saliva═╝/Prostate cancer/pheno.1347_to_prostate cancer_forest.pdf]

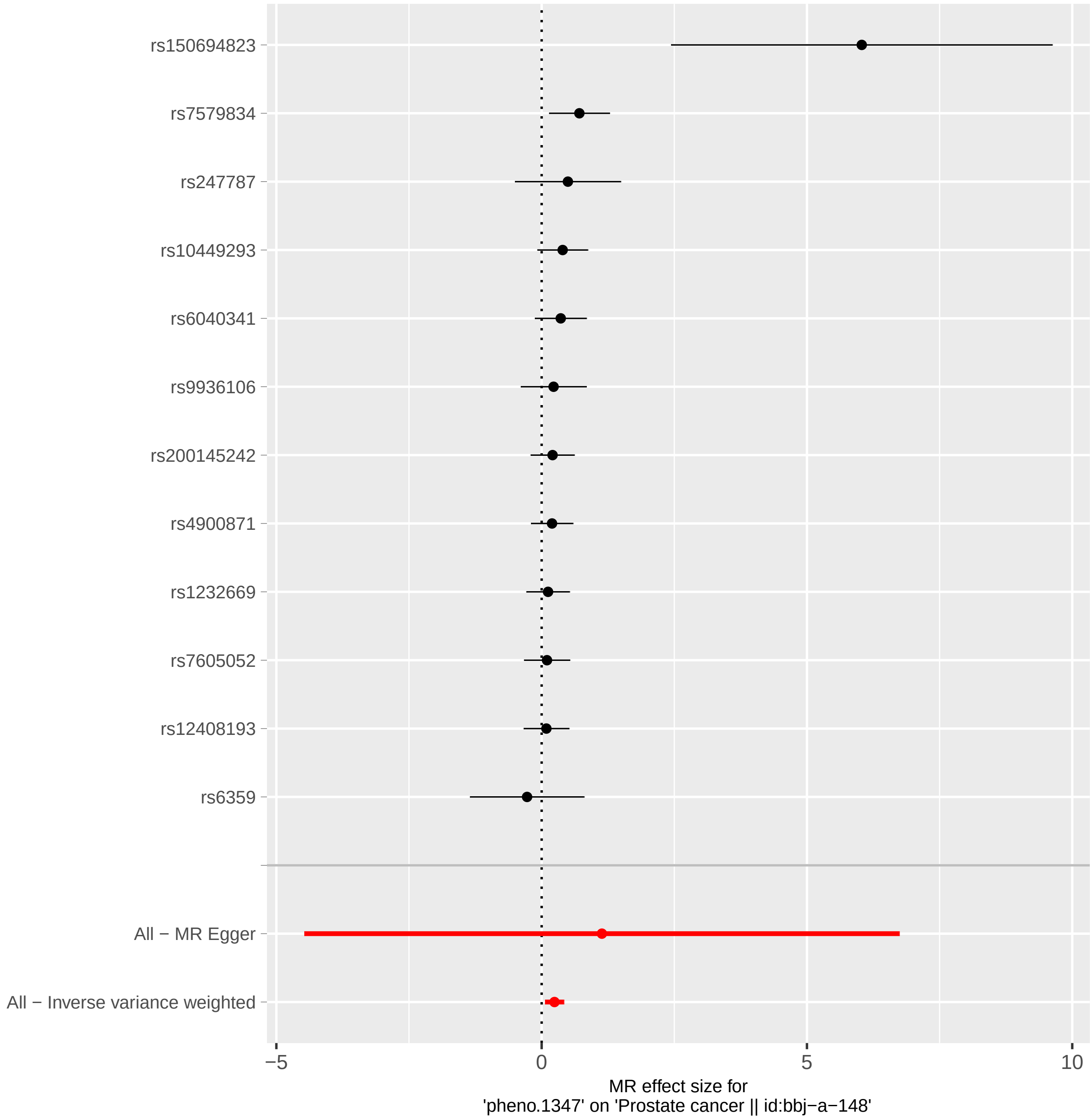

rs150694823
rs7579834
rs247787
rs10449293
rs6040341
rs9936106
rs200145242
rs4900871
rs1232669
rs7605052
rs12408193
rs6359
All − MR Egger
All − Inverse variance weighted
−5
0
5
10
MR effect size for
'pheno.1347' on 'Prostate cancer || id:bbj−a−148'
